# Neurofilament light protein in blood predicts regional atrophy in Huntington disease

**DOI:** 10.1212/WNL.0000000000005005

**Published:** 2018-02-20

**Authors:** Eileanoir B. Johnson, Lauren M. Byrne, Sarah Gregory, Filipe B. Rodrigues, Kaj Blennow, Alexandra Durr, Blair R. Leavitt, Raymund A. Roos, Henrik Zetterberg, Sarah J. Tabrizi, Rachael I. Scahill, Edward J. Wild, for the TRACK-HD Study Group

**Affiliations:** From the Huntington's Disease Research Centre (E.B.J., L.M.B., S.G., F.B.R., S.J.T., R.I.S., E.J.W.), UCL Institute of Neurology, London, UK; Clinical Neurochemistry Laboratory (K.B., H.Z.), Sahlgrenska University Hospital, Mölndal, Sweden; Institut du Cerveau et de la Moelle épinière (A.D.), Sorbonne Universités, UPMC University Paris 06, UMRS 1127, INSERM, U 1127, CNRS, UMR 7225; APHP (A.D.), Genetics Department, Pitié-Salpêtrière University Hospital, Paris, France; Centre for Molecular Medicine and Therapeutics (B.R.L.), University of British Columbia, Vancouver, BC, Canada; Department of Neurology (R.A.R.), Leiden University, the Netherlands; Department of Molecular Neuroscience (H.Z.), UCL Institute of Neurology, Queen Square, London, UK; Department of Psychiatry and Neurochemistry (H.Z.), Institute of Neuroscience and Physiology, the Sahlgrenska Academy at the University of Gothenburg, Mölndal, Sweden; and UK Dementia Research Institute (H.Z.), London, UK.

## Abstract

**Objective:**

Neurofilament light (NfL) protein in blood plasma has been proposed as a prognostic biomarker of neurodegeneration in a number of conditions, including Huntington disease (HD). This study investigates the regional distribution of NfL-associated neural pathology in HD gene expansion carriers.

**Methods:**

We examined associations between NfL measured in plasma and regionally specific atrophy in cross-sectional (n = 198) and longitudinal (n = 177) data in HD gene expansion carriers from the international multisite TRACK-HD study. Using voxel-based morphometry, we measured associations between baseline NfL levels and both baseline gray matter and white matter volume; and longitudinal change in gray matter and white matter over the subsequent 3 years in HD gene expansion carriers.

**Results:**

After controlling for demographics, associations between increased NfL levels and reduced brain volume were seen in cortical and subcortical gray matter and within the white matter. After also controlling for known predictors of disease progression (age and CAG repeat length), associations were limited to the caudate and putamen. Longitudinally, NfL predicted subsequent occipital gray matter atrophy and widespread white matter reduction, both before and after correction for other predictors of disease progression.

**Conclusions:**

These findings highlight the value of NfL as a dynamic marker of brain atrophy and, more generally, provide further evidence of the strong association between plasma NfL level, a candidate blood biomarker, and pathologic neuronal change.

The identification of sensitive biomarkers of disease progression could help in the development of successful treatments for neurodegenerative conditions. Blood biomarkers are particularly appealing as they provide a quick, noninvasive, objective, and reproducible way of quantifying markers of disease progression. Neurofilament light (NfL) has been recognized as a CSF biomarker of neuronal damage for a number of years, but recent advances in ultrasensitive immunoassays have enabled quantification of NfL from blood samples.^1^ Recently, NfL was proposed as a candidate prognostic blood biomarker of Huntington disease (HD) onset and progression.^2^ Baseline NfL was retrospectively quantified in plasma using the well-characterized TRACK-HD cohort.^3–6^ NfL was increased in HD gene expansion carriers when compared to controls, with NfL levels reflecting baseline motor and cognitive deficits, as well as reduced global and regional brain volume. Baseline NfL also predicted disease progression in these 3 domains over the following 3 years. This previous study highlights the potential importance of NfL as a promising dynamic blood biomarker of not only current disease status and onset, but also ongoing progression in HD. However, global or region-of-interest measures provide limited insight into the regional distribution of NfL-associated pathology due to the HD gene mutation. Here, we use both cross-sectional and longitudinal voxel-based morphometry (VBM) in the same TRACK-HD cohort to map the specific relationship between NfL and progressive regional atrophy.

## Methods

### Participants

Participants were recruited as part of the multisite longitudinal TRACK-HD study, which included 120 participants with premanifest HD (preHD) and 123 participants with manifest HD.^2^ Participants were recruited in 2008 from hospital clinics at 4 sites based in Leiden, London, Paris, and Vancouver and were classified as having premanifest or manifest HD at baseline depending on their score on the Unified Huntington's Disease Rating Scale (UHDRS) Total Motor Score.^7^ A score of ≤5 meant that a participant was categorized as preHD, and participants with a Total Motor Score >5 combined with a UHDRS Total Functional Capacity score >7 were classed as early manifest HD. Disease Burden Score,^7^ an approximate marker of disease load, and 5-year probability of onset,^8^ an approximate prediction of disease onset, were calculated for this cohort. Participants underwent a series of assessments and returned yearly until 2011. Sample size was calculated before the TRACK-HD baseline visit and was aimed at detecting significant longitudinal change over 2 years in different variables.^3^ Full study and recruitment information has been documented previously.^3^

### Standard protocol approvals, registrations, and patient consents

Local ethical approval was given for the study and all participants gave their written informed consent according to the Declaration of Helsinki.

### Imaging acquisition and processing

Three-tesla MRI scans were collected at all 4 sites using a standardized T1-weighted acquisition developed for this study.^2^

Scans underwent rigorous quality control, with meta-data checks and visual quality-control steps performed to ensure that acquisition parameters were correct and to exclude scans with motion and other artifacts. All scans that passed quality control were then processed using SPM 12 (http://www.fil.ion.ucl.ac.uk/spm/) with MATLAB version R2012B (https://in.mathworks.com). Baseline volumes were separated into different tissue classes (gray matter [GM], white matter [WM], and CSF) using the Segment procedure. A group template was created using Diffeomorphic Anatomical Registration Through Exponentiated Lie Algebra^10^ (DARTEL), and the WM and GM tissue classes were then warped to the DARTEL template. These images were modulated and smoothed to account for any volume changes that occurred during normalization. A 4-mm kernel at full-width half maximum was used for smoothing. All images were visually examined to check segmentation and normalization performance.

Longitudinal VBM was performed as described previously.^5,11^ Within-subject longitudinal change was first measured using a nonlinear fluid registration technique within in-house MIDAS software.^12^ Voxel-compression maps were generated for each participant from baseline to 3-year follow-up.^11,13^ These voxel-compression maps were then spatially mapped onto the DARTEL template and then convolved with the participant-specific baseline GM and WM maps to generate voxel-level, within-subject change for each tissue type.^11^

Total intracranial volume was measured via the MIDAS software^12^ using a semiautomated procedure as previously described.^14^ All image analyses were performed blinded to participant group to avoid potential sources of bias.

### NfL quantification

NfL was quantified in plasma samples using an ultrasensitive single-molecule array method as previously described.^6^ All samples were analyzed in one round of experiments using one batch of reagents. All NfL values were within the linear ranges of the assays, and intra-assay coefficients of variation were below 10%. NfL quantification was performed blinded to group to avoid potential sources of bias.

### Statistical analysis

Associations between NfL and (1) cross-sectional GM or WM volumes, and (2) longitudinal GM and WM change were examined using linear regression models within SPM 12. Age, sex, study site, and total intracranial volume were initially controlled for in the model, to permit identification of regions where atrophy was associated with plasma NfL. To identify regions where NfL independently predicted atrophy, after adjustment for known predictors of HD progression, the analysis was then repeated with CAG repeat length and age–CAG interaction as terms. These terms are known predictors of HD disease onset and stage, and thus by controlling for these factors, we can better understand the independent influence of NfL on GM and WM.^15^ Explicit binary masks for GM and WM were used in the analysis, as described previously.^16^ All results were one-tailed and corrected for multiple comparisons at *p* = 0.05 using family-wise error.

## Results

Of the 243 gene expansion carriers recruited in TRACK-HD, 201 had plasma samples collected at baseline (2008) from which NfL could be measured, and 198 also had structural imaging that passed quality control at baseline. Of these participants, 176 had both plasma samples and structural MRI scans at the 3-year follow-up (2011), with an additional participant who had a structural MRI scan but no plasma. Although retention was high for the study, the most common reason for drop-out at the 2011 time point was worsening of symptoms. Detailed information on retention was discussed previously.^3^ Demographic information is provided in the table.

**Table:**
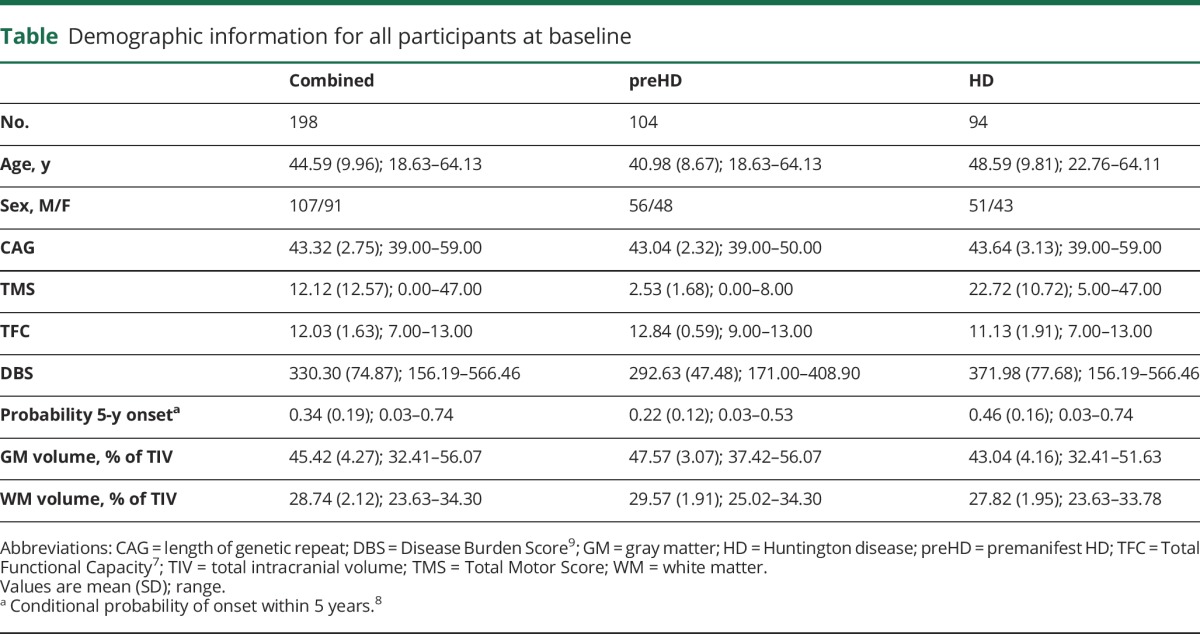
Demographic information for all participants at baseline

### Baseline NfL: Relationship to GM and WM volume at baseline

**Figure 1:**
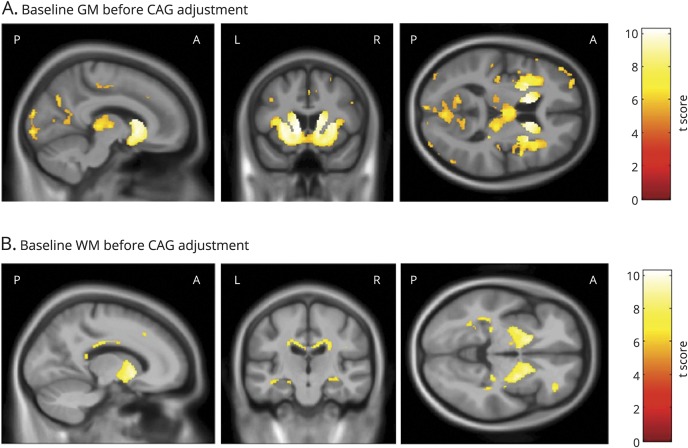
Significant associations between baseline NfL and baseline GM and WM volume Statistically significant negative associations between baseline NfL and (A) baseline GM volume and (B) baseline WM volume. All analyses were adjusted for age, sex, study site, and total intracranial volume corrected at *p* < 0.05 family-wise error and are displayed on a study-specific template. GM = gray matter; NfL = neurofilament light; WM = white matter.

**Figure 2:**
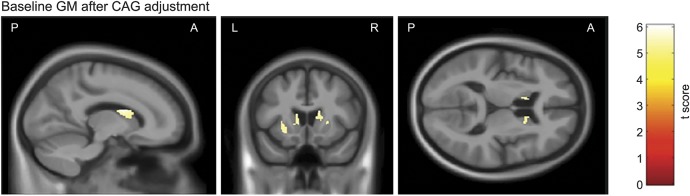
Significant associations between baseline NfL and baseline GM volume after adjustment for CAG Statistically significant negative associations between baseline NfL and baseline GM volume after additional adjustment for CAG repeat length and the interaction between age and CAG repeat length. All analyses were adjusted for age, sex, study site and total intracranial volume corrected at *p* < 0.05 family-wise error and are displayed on a study-specific template. GM = gray matter; NfL = neurofilament light.

Before adjusting for CAG repeat length and the CAG × age interaction, there were significant associations between higher baseline plasma NfL and lower subcortical and cortical volume of the GM, especially within the striatum and occipital lobe (figure 1A). Significant associations were also seen with reduced WM surrounding the striatum and within the corpus callosum (figure 1B). Following adjustment for CAG repeat length and the interaction between age and CAG repeat length, higher baseline NfL was significantly associated with reduced volume in the caudate and putamen bilaterally (figure 2). There were no associations between NfL and WM volume that survived correction for multiple comparisons.

### Baseline NfL: Relationship to longitudinal GM and WM volume change

**Figure 3:**
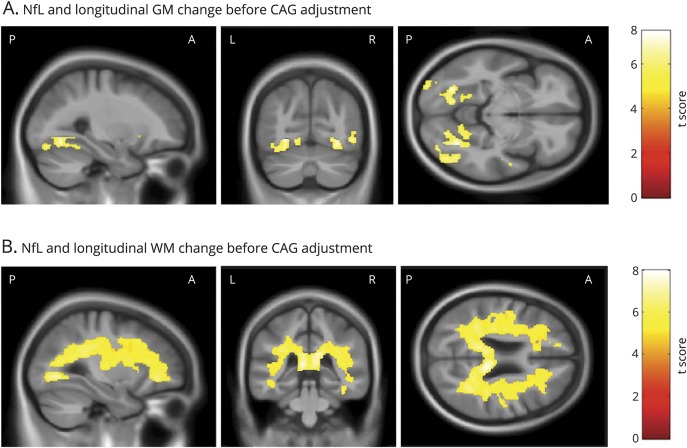
Significant associations between baseline NfL and change in GM and WM volume Statistically significant negative associations between baseline NfL and longitudinal change in (A) GM volume and (B) WM volume. All analyses were adjusted for age, sex, study site, and total intracranial volume, corrected at *p* < 0.05 family-wise error, and are displayed on a study-specific template. GM = gray matter; NfL = neurofilament light; WM = white matter.

**Figure 4:**
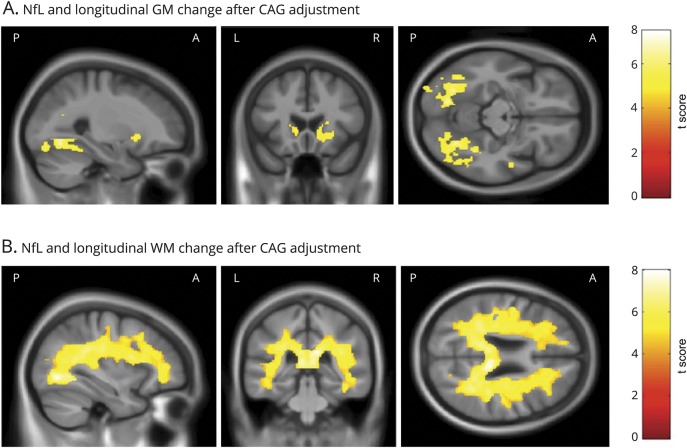
Significant associations between baseline NfL and change in GM and WM volume after adjustment for CAG Statistically significant negative associations between baseline NfL and longitudinal change in (A) GM volume and (B) WM volume, after additional adjustment for CAG repeat length and the interaction between age and CAG repeat length. All analyses were adjusted for age, sex, study site, and total intracranial volume corrected at *p* < 0.05 family-wise error and are displayed on a study-specific template. GM = gray matter; NfL = neurofilament light; WM = white matter.

Higher baseline levels of plasma NfL were positively associated with subsequent longitudinal volume loss bilaterally in the caudate, putamen, and occipital cortex (figure 3A). Higher baseline NfL was also positively associated with subsequent widespread volume reduction within the WM, most significantly in the corpus callosum (figure 3B). Figure 4A shows that after controlling for CAG repeat length and the interaction between age and CAG repeat length, NfL primarily predicted subsequent GM reduction within the inferior occipital cortex and the lateral occipital cortex. Significant associations were also seen in small regions within the inferior frontal gyrus and superior temporal gyrus. Finally, higher baseline NfL again showed widespread associations with longitudinal WM volume reductions, even after adjusting for CAG and the age × CAG interaction term (figure 4B).

## Discussion

This study presents unbiased whole-brain results showing voxel-wise region-specific associations between NfL levels measured in plasma and both cross-sectional and longitudinal MRI GM and WM volume in a pathologic group previously shown to have raised NfL levels. One previous study in HD found associations between the level of NfL in plasma and MRI measures of cross-sectional brain volume and brain atrophy over the subsequent 3 years in a number of predefined regions of interest using global values.^6^ Here, we build on this previous work, using VBM to reveal the location and extent of volumetric change in brain regions that are associated with NfL levels in HD.

As expected, higher NfL in plasma was associated with lower volume in regions known to be affected in HD. After adjustment for CAG repeat length and its interaction with age—known predictors of HD progression^15^—NfL remained independently associated with reduced GM volume in the caudate and putamen bilaterally, but there was no association with cortical or WM volume at baseline. The caudate and putamen are regions that undergo the earliest and most extensive atrophy in HD due to the predominance of medium spiny neurons that are particularly vulnerable to the effects of mHTT.^15^ However, it appears that baseline NfL level does not specifically relate to more widespread neuronal damage at a given time point, beyond the overall general effect of age and CAG repeat length on neurodegeneration. That the caudate and putamen were associated with NfL concentration after age and CAG adjustment suggests that NfL is capable of identifying individuals who, over the course of their life up to the time of sampling, have experienced relatively larger or smaller volume loss in these disease-linked brain regions.

It is of interest that there was a much stronger relationship between baseline NfL levels and subsequent regional volume loss over time, with these relationships remaining highly significant after adjusting for CAG repeat length and its interaction with age. Higher baseline levels of NfL significantly predicted increased atrophy in cortical but not subcortical regions of the GM over the following 3 years, which is consistent with the previous study reporting stronger association between baseline NfL and total GM than caudate atrophy.^6^ Our results extend this finding by providing further evidence that, after controlling for overall age and CAG effects, higher NfL is predictive of HD gene expansion carriers who will subsequently undergo greater GM atrophy extending beyond the striatum. This study demonstrates that the most significant associations between NfL and subsequent neuronal change are localized to the occipital lobe, one of the earliest cortical regions thought to undergo atrophy in HD.^2,17,18^ While previous studies have found that the occipital lobe appears to be an early region of neural change, neuropathology studies have found that in more advanced cases of HD, cortical atrophy is globally distributed.^19^ The significant association between higher NfL and subsequent atrophy suggests that NfL is sensitive to some of the earliest cortical atrophy in HD.

In addition to cortical GM atrophy, there was a strong relationship between higher baseline NfL levels and widespread atrophy within the WM. WM has been shown to undergo change in both preHD and manifest HD,^5^ and is strongly related to disease progression.^3^ Our findings suggest that, similarly to GM, NfL is predictive of subsequent WM atrophy. These associations indicate that it in addition to its value as an accessible indicator of global neurodegeneration, NfL may have particular value as a marker of axonal degeneration—and therefore potentially reversible neuropathology in regions vulnerable to damage in HD.^20,21^

Whole-brain analyses using VBM have only been used to examine the relationship between NfL—measured in CSF, not plasma—and neuronal change in a neurologic illness (frontotemporal dementia) in one previous study.^22^ However, the current findings are the first to show significant associations between NfL in plasma across the whole brain in both cross-sectional and longitudinal data. Even though plasma NfL has been shown to increase longitudinally in HD, and the associations of such longitudinal change may be of interest,^6^ our results suggest that a single baseline measurement of NfL has the ability to identify gene expansion carriers who have previously undergone disproportionate atrophy in the basal ganglia, and those who are likely to go on to develop similarly disproportionate atrophy in cortical and WM regions vulnerable to HD pathology. This corroborates previous findings^6^ and supports the hypothesis that NfL is a dynamic, rapidly-assessed marker of ongoing neuronal damage. Previous reports from the TRACK-HD study demonstrated that patients with the highest atrophy rates were those who subsequently showed the greatest clinical decline.^3^ Here, we demonstrate that the region-specific predictive power of NfL extends beyond predefined large volumes of interest. This provides further evidence for the utility of NfL as a potential efficacy and disease progression biomarker in therapeutic intervention trials.

We showed a highly significant and widespread association between plasma NfL and change in WM volume using VBM. In the future, use of microstructural measures of WM degeneration derived from diffusion imaging may allow a detailed characterization of the relationship with NfL, providing further mechanistic insights into the breakdown of brain connectivity, which we know is a key feature of HD.^20,23^ Because the progression of neuronal atrophy beyond the caudate in preHD and manifest HD is fairly slow, analysis using longer time intervals and a wider range of disease stages would be useful to further establish the relationship between NfL and neurodegeneration. Application of these techniques in other disease cohorts will help us to understand the wider role of NfL in the neurodegenerative process. Furthermore, this study is limited because the analysis was performed on a group of HD gene expansion carriers across a range of premanifest and manifest stages. This provides more power to detect effects; however, further analyses within subgroups could provide detailed information on different stages of the disease. Finally, because of individual differences among participants, our findings cannot be applied meaningfully to individual HD mutation carriers or for clinical decision-making.

We provide further evidence supporting the use of NfL as a prognostic marker of progression of neuronal damage in both HD and other neurodegenerative diseases. NfL appears to be a significant indicator of subsequent widespread brain changes extending beyond the striatum, particularly within the WM. The ability to measure NfL from plasma provides an easily accessible biomarker that has close links to the underlying pathology of HD and shows promise as a dynamic marker of ongoing neuronal change.

## Glossary

DARTEL: Diffeomorphic Anatomical Registration Through Exponentiated Lie Algebra
GM: gray matter
HD: Huntington disease
NfL: neurofilament light
preHD: premanifest Huntington disease
UHDRS: Unified Huntington's Disease Rating Scale
VBM: voxel-based morphometry
WM: white matter
